# Beyond mechanics: Maximum‐likelihood‐driven PET detector alignment calibration

**DOI:** 10.1002/mp.70256

**Published:** 2025-12-31

**Authors:** Julian Thull, Yannick Kuhl, Florian Mueller, David Schug, Bjoern Weissler, Volkmar Schulz

**Affiliations:** ^1^ Department of Physics of Molecular Imaging Systems RWTH Aachen University Aachen Germany; ^2^ Chair of Imaging and Computer Vision RWTH Aachen University Aachen Germany; ^3^ Hyperion Hybrid Imaging Systems GmbH Aachen Germany; ^4^ Fraunhofer Institute for Digital Medicine MEVIS Bremen Germany

**Keywords:** detector alignment, in‐system calibration, PET

## Abstract

**Background:**

Accurate quantification is a cornerstone of positron‐emission tomography (PET), yet mechanical inaccuracies during detector assembly continue to undermine this goal. Deviations from intended design geometries arise due to factors such as manufacturing tolerances, mechanical stress, and cumulative errors during module installation and operation. High‐resolution PET systems, in particular, are highly susceptible to even minor misalignments, such as translational offsets of 0.5mm and angular deviations of 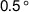, leading to distorted lines of response, a reduction in signal‐to‐noise ratio, and compromised quantitative reliability.

**Purpose:**

To address the limitations imposed by mechanical misalignments in PET systems, particularly in high‐resolution applications, we propose a calibration strategy that eliminates reliance on precise physical assembly or movable calibration setups. The goal is to develop a data‐driven framework for estimating detector alignment parameters directly from PET measurements, allowing for robust recalibration across a broad range of scanner geometries and detector designs.

**Methods:**

We present a statistical optimization framework that estimates detector alignment parameters directly from time‐of‐flight list‐mode coincidence data using gradient‐based optimization. By casting the problem as a maximum likelihood estimation conditioned on a known 3D tracer distribution, the optimization targets to maximize the Poisson likelihood of the measured coincidence data under the parametrized alignment model. This formulation eliminates the need for any movable parts, including motion stages, rotating sources, or manual calibration efforts. The approach supports complex extended tracer distributions and generic scanner geometries, enabling a flexible, regular, software‐based recalibration from static acquisitions.

**Results:**

The method was validated on both simulated and real PET systems, including measurements with point sources and a tracer‐filled tube phantom. In the simulation study, deviations from a cylindrical configuration were introduced prior to data generation, while the cylindrical geometry was used as initialization for the optimization. For the point source setup, alignment parameters were recovered with an accuracy of approximately 50μm and 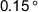, and for the simulated tube phantom, with approximately 120μm and 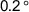. On two real scanners with differing crystal topologies and arbitrarily induced misalignments to the blueprint initialization, the recovered configurations yielded image resolutions in the range of 1.0mmto1.2mm, matching the performance of a precisely known design specification. For both real systems, the optimizations relied on approximately five million coincidences, demonstrating that accurate alignment can be achieved with modest amounts of measurement data. Notably, for the second scanner, a single gamma positioning model was shared across all detectors, indicating the method's robustness towards model‐sharing constraints.

**Conclusions:**

This framework bridges hardware imperfections and quantitative fidelity by enabling robust recalibration in mechanically unstable or mobile settings. It is compatible with diverse phantom types and scanner topologies, and operates effectively even in the absence of precise scanner‐phantom alignment. By reducing dependency on strict manufacturing tolerances while preserving an accurate line of response positioning, our approach offers a practical and scalable solution for modern PET calibration, where quantitative accuracy is paramount.

## 1 INTRODUCTION

Tomographic image reconstruction is based on the fundamental relationship between integral values along certain manifolds and real‐world measurements acquired by tomographic imaging devices.^1^ In positron‐emission tomography (PET), these manifolds correspond to lines of response (LOR), which represent the paths along which gamma‐ray pairs are detected. These gamma pairs originate from the β+ decay of a radioactive tracer and the subsequent annihilation of emitted positrons with nearby electrons, producing two photons traveling in nearly opposite directions. The detection of these gamma photons by scintillation crystal arrays enables the parametrization of each coincidence event as a LOR in 3D, intersecting the distribution of the radioactive tracer. Time‐of‐flight (TOF) information additionally localizes the tracer position along the LOR. Spatial integration along these TOF‐weighted LOR manifolds then serves as expected measurement model for a PET scanner (up to corrections) and yields the backbone for tomographic PET image reconstruction algorithms.

In practice, the 3D spatial precision of each LOR ultimately limits the biologically relevant detail a clinical PET system can reveal. This is particularly relevant for high‐resolution systems, such as those used in neuroimaging, where early detection of Alzheimer's disease depends on the ability to visualize pathological markers like amyloid plaques at sub‐millimeter resolution.^2^ Achieving this high level of precision depends not only on advanced detector design and calibration but also on the precision of their mechanical alignment. Imprecise estimates of detector positions and orientations lead to the systematic misplacement of LORs and consequently to a reduced image quality. Although advancements in material sciences and manufacturing methods have facilitated the creation of custom mounting structures, the adoption of PET/MR systems, and the implementation of non‐standard scanner geometries make it more challenging to achieve the mechanical precision of conventional systems. At the same time, the pursuit of sub‐millimeter image resolution imposes stricter requirements on the precision of the mechanical mounting. In response, software‐based in‐system calibration has been put forward by the PET community,^3^, ^4^, ^5^, ^6^, ^7^, ^8^ enabling the estimation of detector block alignment parameters by a measurement‐based calibration routine. Related works use a calibration based on statistical and trigonometric properties of the measured data and involve single point source measurements for which precise positron–electron annihilation locations are always known. Also, these approaches do not exploit time‐of‐flight information for optimization, which can be used to further improve calibration accuracy. Despite achieving promising results, long measurement times, calibration of motor stages, and the need for manual user inputs present ongoing challenges that need to be addressed. This is especially relevant in clinical environments where throughput and scheduling are critical, emphasizing the need for fast and simple calibration routines that can be seamlessly integrated into clinical workflow.

Pierce et al.^3^ conducted a simulation study using a single point source moved in a circular path while being incrementally shifted along the *z*‐axis after each full rotation. For each point source location a triangulation for the corners of the scintillation crystals was performed, yielding positional estimates for all detector blocks. Later, Pierce et al.^4^ followed up on this by extending the procedure to cover all six degrees of freedom of the detector blocks. They reported results in terms of the Euclidean distance d and cylindrical coordinates r, θ, and z, and achieved an average detector positioning accuracy of d=0.62mm, with mean component errors of r=0.48mm, $\theta =0.12^{\circ }$, and z=0.26mm. Abella et al.^5^ performed a detailed analysis of the effects of detector misalignment on sinograms and presented a calibration method based on two acquisitions of a 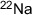 point source, positioned at the center of the field of view (FOV) and off‐centered. The method did require additional user input to fine‐tune the calibration estimate. They reported mean positioning errors of x=1.07mm, y=0.40mm, z=0.30mm, and an angular error of $0.42^{\circ }$ after calibration. Sabet et al.^6^ proposed a calibration protocol in which “ideal” LORs are defined as the lines connecting the centers of opposing detector block crystals. Point source measurements were performed on two lines perpendicular to the ideal LORs. The detector block positions were then optimized to minimize deviations in the LOR intersection points. They applied their detector coordinate correction method to a real system, achieving an improvement in spatial resolution from ${\mathrm{SR}}_{x}=1.22\;\mathrm{mm}$ and ${\mathrm{SR}}_{y}=1.42\;\mathrm{mm}$ to ${\mathrm{SR}}_{x}=0.87\;\mathrm{mm}$ and ${\mathrm{SR}}_{y}=1.07\;\mathrm{mm}$.

Klopping et al.^7^ used the Millipede‐II algorithm^9^ to perform alignment for all crystals in a PET/MR insert device. They reported accurate recovery of misalignments using a single point source for data acquisition. They demonstrated, additionally, with simulated data that image reconstructions using uncorrected geometries showed significant degradation compared to those obtained after applying the track‐based alignment optimization. Matthews and Wang^8^ presented a patented calibration method for coincidence imaging systems, wherein the distance between each positioned LOR and a known point source position was computed and minimized. The optimization adjusted detector positions and orientations so that all LORs intersected the known point source locations with high precision. All studies addressed the problem of misalignment by proposing optimization techniques that require the supervision of the positron–electron annihilation location for each detected coincidence.

We build upon this foundation by introducing an approach that frames detector alignment calibration as a distribution matching problem, optimizing alignment parameters via maximum likelihood estimation. Unlike previous methods, our approach accommodates complex tracer distributions and leverages time‐of‐flight information, enabling a more flexible and accurate calibration process. The proposed approach optimizes rotations and translations of detector blocks to maximize the likelihood of observing the measured TOF‐binned list‐mode counts from the ground‐truth phantom definition and precomputed detection efficiencies. This approach presupposes gamma positioning (3D or planar only) and timing calibration of the detectors. Standard segmented crystals provide only planar positioning, whereas semi‐monolithic detectors also deliver DOI information. The proposed calibration works in both configurations, though performance improves when DOI information is available. Notably, our calibration procedure aligns well with the optimization performed in iterative image reconstruction, where the Poisson likelihood is similarly optimized to refine image estimates to match the measured data. Due to its formulation in this framework, the method enables the use of dedicated calibration phantoms instead of relying on single point source measurements or carefully arranged multiple point sources with separable LORs, thereby eliminating the need for motor stages and their calibration,^10^, ^11^, ^12^ as well as strict source separability. We find that our approach delivers promising results for simulated and real scanners, evaluated by the translational and rotational deviations as well as the produced image quality.

In the following, we introduce the general framework for maximum likelihood‐based alignment estimation of PET detector blocks. This is followed by a description of the modeling approach, implementation aspects, and the simulation and measurement setups. Results are presented for a simulated PET scanner, a real system using semi‐monolithic slab detectors, and another real system employing multi‐layer segmented crystal arrays. Finally, we discuss practical implications and limitations, and conclude with a summary of the study.

## 2 MATERIALS AND METHODS

### 2.1 Alignment modeling

A PET system consists of $N_{\text{det}}$ detector blocks arranged according to a known design geometry (blueprint), where the detectors have defined positions $P\in R^{N_{\text{det}}\times 3}$ and axes configurations $X,Y,Z\in R^{N_{\text{det}}\times 3}$. Such blueprints often represent a cylindrical arrangement in order to maximize sensitivity. However, due to mechanical imprecisions, tolerances, and stress, actually manufactured scanners $P^{*},X^{*},Y^{*},Z^{*}$ (which we will refer to as ground‐truth) deviate from this alignment by unknown rotations and translations $R,T\in R^{N_{\text{det}}\times 3}$ of detector blocks. Given the blueprint configuration, the predicted alignment is given by:

(1) $$\begin{array}{cc} {\hat{P}}_{d} & =P_{d}+T_{d} \end{array}$$

(2) $$\begin{array}{cc} \left[{\hat{X}}_{d}\;{\hat{Y}}_{d}\;{\hat{Z}}_{d}\right] & =R_{X_{d},Y_{d},Z_{d}}\left(R_{d}\right)\;\left[X_{d}\;Y_{d}\;Z_{d}\right]. \end{array}$$

**FIGURE 1:**
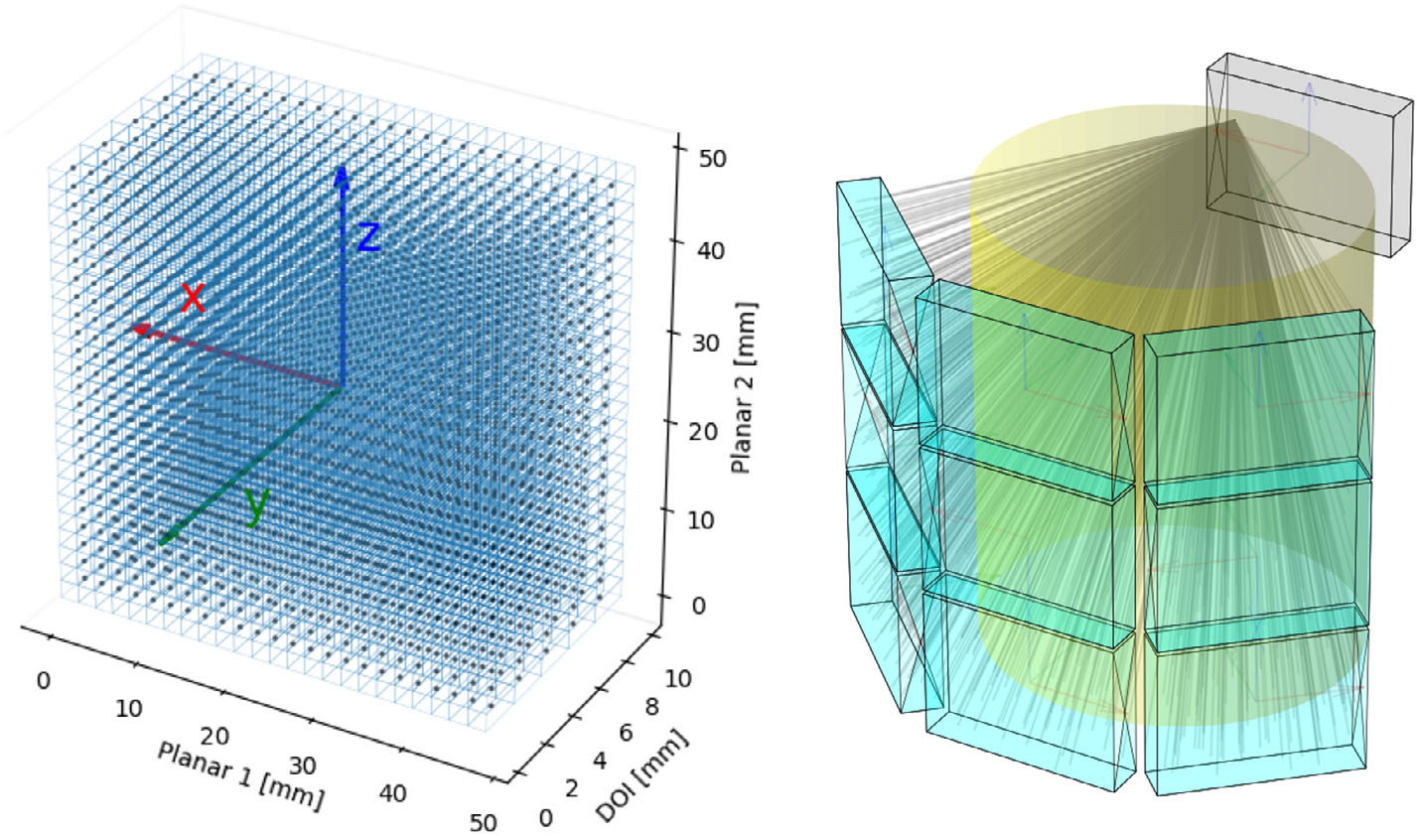
Left: Visualization of lookup table L for a crystal binning of 24×24×5 crystal voxels per block, resulting in a $2\times 2\times 2\;mm^{3}$ volume per voxel. Crystal voxel centers are marked in black, and their spatial bounds are shown in blue. Coordinate axes are overlaid in red (planar *x*‐axis), green (DOI *y*‐axis), and blue (planar *z*‐axis). Right: Coincidence acceptance matrix for fan‐sum efficiency computation under cylindrical shell phantom acquisition with a random subset of accepted LORs.

The optimization seeks rotations R and translations T so that the transformed blueprint $\left({\hat{P}}_{d},{\hat{X}}_{d},{\hat{Y}}_{d},{\hat{Z}}_{d}\right)$ matches with the ground‐truth configuration $\left(P_{d}^{*},X_{d}^{*},Y_{d}^{*},Z_{d}^{*}\right)$. Rotation matrix $R_{X_{d},Y_{d},Z_{d}}\left(R_{d}\right)$ denotes the rotation by angles $R_{d}$ of a vector about the local axes $X_{d},Y_{d},Z_{d}$ of detector block d. Accurate LOR placement also depends on the localization of interaction events within each block, as determined by crystal identification algorithms, that is, gamma positioning calibration models. For this, we assume each interaction is assigned to a crystal voxel, and represent voxel centers in the local detector coordinate system via a lookup table $L:N_{\text{cr}}\to R^{3}$, where $N_{\mathrm{cr}}$ denotes the number of crystal voxels per block, cf. Figure 1 (left). Additionally, the method supports regular and irregular crystal layouts with non‐uniform spacing, and enables sampling within individual voxel bounds. This is particularly useful for light‐sharing detector designs, where spatial encoding must account for varying voxel dimensions. The transformation of a crystal voxel center λ=(d,cr), where $d\in [0,N_{\text{det}}-1]$ and $cr\in [0,N_{\text{cr}}-1]$, into global coordinates is given by:

(3) $$\begin{array}{cc} p_{R,T}\left(\lambda |P,X,Y,Z\right) & =\hat{P_{d}}+L{\left(cr\right)}_{x}\cdot \hat{X_{d}}\\ & \;+L{\left(cr\right)}_{y}\cdot \hat{Y_{d}}+L{\left(cr\right)}_{z}\cdot \hat{Z_{d}}. \end{array}$$

### 2.2 Detection efficiency

Each crystal voxel λ has an associated detection efficiency $E_{\lambda }$, reflecting the probability that a gamma photon is detected, given that it has interacted within the voxel. These efficiencies account for effects such as optical coupling, crystal imperfections, positioning biases, and contributions from individual Silicon Photomultipliers (SiPMs), and are defined relative to the ideal interaction model. We estimate these efficiencies in‐system using the fan‐sum method, a technique commonly used under the assumption of a cylindrical scanner geometry.^13^ For this purpose, we use measurements acquired with a cylindrical shell phantom (for scanner no. 1, cf. Figure 1 (right)) and a line source (for scanner no. 2), respectively. For each crystal voxel, we count the number of coincidence events detected within a fixed time span and within an energy window, yielding unnormalized detection counts. These raw counts are used directly as efficiency estimates. No additional geometric corrections are applied, as these would require prior knowledge of the actual scanner alignment. While geometry‐related sensitivity variation—due to limited axial coverage, detector gaps, and angular fan‐beam effects—can be substantial even in idealized designs, we find that for moderate misalignments, such uncorrected counts provide a sufficiently robust efficiency approximation for alignment optimization. Consequently, the estimated efficiencies remain approximations and must be recomputed after alignment optimization for accurate use in image reconstruction. For scanner geometries that deviate substantially from a cylindrical blueprint by design, efficiency estimation requires detector‐individual benchtop flood measurements to obtain count‐based efficiency estimates that are fully independent of the system geometry.

### 2.3 LOR modeling

Coincidence events consist of two crystal bins $\lambda_{1},\lambda_{2}$ and a TOF bin t, derived from the time difference of photon arrivals. The expected counts for all $\left(\lambda_{1},\lambda_{2},t\right)$ bins are proportional to the tracer line integrals, corrected by LOR detection efficiency and attenuation. For each bin, we compute line integrals in the attenuation image along the full line of response, while in the tracer image, the integral is weighted by the Gaussian TOF kernel $K_{\text{TOF}}$, whose width is determined by the scanner's timing resolution. Given a known tracer distribution F, attenuation map A, both represented as 3D voxel grids, and per‐crystal detection efficiencies E, the expected measurement model is defined as:^14^

(4) $$\begin{array}{cc} L(\lambda_{1},\lambda_{2},t)= & \;E_{\lambda_{1}}E_{\lambda_{2}}e^{-\int_{p(\lambda_{1})}^{p(\lambda_{2})}A\left(\gamma \left(l\right)\right)\;dl}\\ & \cdot \int_{p(\lambda_{1})}^{p(\lambda_{2})}K_{\text{TOF}}\left(\gamma \left(l\right),t\right)F\left(\gamma \left(l\right)\right)\;dl. \end{array}$$

Here, the integration follows the straight‐line path parameterized by l, where γ(l) denotes the spatial position along the path between positions $p(\lambda_{1})$ and $p(\lambda_{2})$. No scatter component is modeled in this formulation, though it could be incorporated in future extensions if a differentiable approximation is available. The actually measured data of a PET scan is represented as a coincidence histogram $C(\lambda_{1},\lambda_{2},t)$, which counts the number of detected coincidences per bin.

### 2.4 Likelihood optimization

The goal of maximum likelihood alignment optimization is to determine the ground‐truth scanner configuration in terms of deviations R,T from the blueprint. This is achieved by maximizing the likelihood of observing the measured counts under the R,T‐parametrized expectation model. The expected measurement distribution is matched to the observed coincidences $C(\lambda_{1},\lambda_{2},t)$ under the Poisson likelihood model:

(5) $$\begin{array}{cc} \left(R,T\right)=\arg\min_{R,T}E_{(\lambda_{1},\lambda_{2},t)∼C} & \left[C\left(\lambda_{1},\lambda_{2},t\right)\log L\left(\lambda_{1},\lambda_{2},t\right)\right.\\ & \left.\;-L(\lambda_{1},\lambda_{2},t)\right]. \end{array}$$

We optimize this function for 200 iterations using the Adam optimizer^15^ and sample a random batch of 5 million crystal bin combinations and TOF bins. For each combination, we sample five positions inside the crystal voxel bounds of both crystals and utilize the mean line integral for the optimization. We choose 0.1 and 1 as learning rates for R and T, respectively, and otherwise use the standard Adam parameters. We use a voxel size of 0.125mm for tracer and attenuation images. The complete optimization is implemented in python using the pytorch framework.^16^ We utilize the parallelproj package^17^ for fast line integral computations on the GPU using Joseph's method.^18^ All computations were performed on an NVIDIA RTX 6000 Ada GPU, requiring approximately 1 h 40 min for the optimization and a maximum of only 16 GB of VRAM.

### 2.5 Gate simulation setup

**FIGURE 2:**
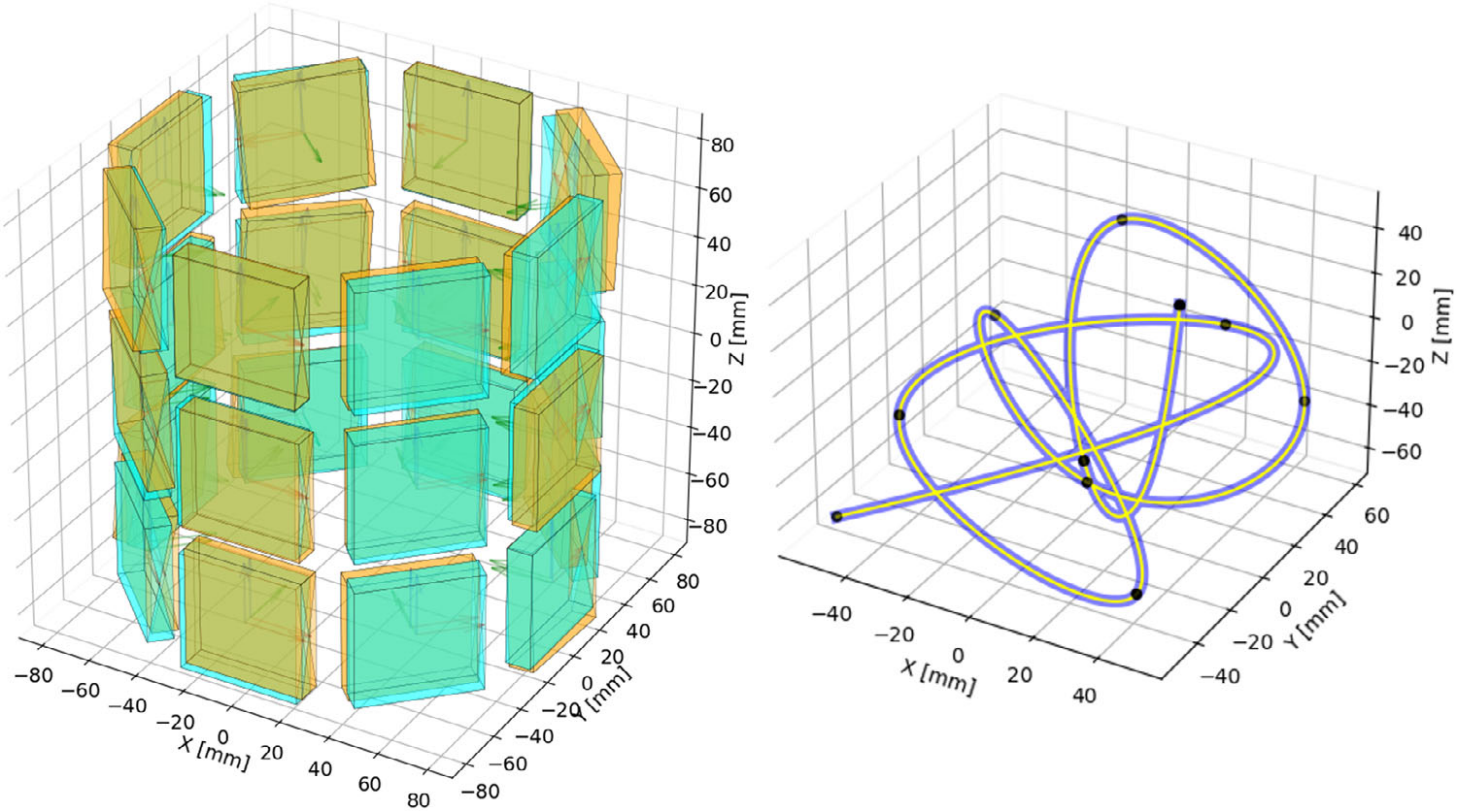
Left: Initial cylindrical configuration (blue) and ground‐truth geometry (orange) used for simulation. Right: Visualization of point source arrangement (black) and resulting B‐spline tube phantom (blue/yellow) in 3D.

We evaluate our method with respect to a simulated small‐animal PET‐scanner using the open‐source software Gate v9.2.^19^ The simulated scanner features 24 detector blocks of 8 detectors arranged in 3 rings at an approximate inner radius of 75mm. The detector blocks have a size of $48\times 48\times 10\;mm^{3}$. Deviations from a cylindrical setup were applied by adding Gaussian noise to the positions and axes (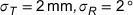), which were integrated into the simulation using Gate's generic repeater functionality. Cylindrical and randomized setups are visualized in Figure 2 (left). For digitization we utilized and energy‐weighted centroid across crystals, an energy resolution of 12%, a CTR of 250ps, and a 5ns coincidence timing window. For this scanner we simulated $10\times^{22}\mathrm{Na}$ point sources randomly distributed within the field of view (FOV). Each source has a radius of 0.125mm and is embedded within an acrylic glass cube (PMMA) measuring 1×1× $1\;mm^{3}$. 42 585 039 coincidences were acquired after digitization.

Additionally, we simulated a tube phantom with a diameter of 250μm, resembling an infusion tube filled with 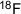
‐FDG (cf. Figure 2, right). In practice, the tube could be fixed in place and first filled with a computed tomography (CT) or magnetic resonance (MR) contrast agent to characterize the tracer distribution geometry. These imaging modalities serve only to obtain a high‐resolution 3D reference of the expected activity distribution prior to the PET experiment, not to figure out the position of the phantom. After this initial imaging step, the contrast agent would be replaced with 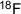
‐FDG, and the phantom would subsequently be used for the PET acquisition. This procedure does not require a combined PET/CT or PET/MR system, as the scans can be performed sequentially. For the simulation, we utilized the back‐to‐back emission type, which substantially reduced simulation runtime compared to a voxelized attenuation approach with fine voxel resolution. This simplification comes at the expense of neglecting non‐collinearity and positron range effects. The 10 previously defined point sources serve as control points for a B‐spline, defining the spatial distribution of activity along the tube. Both phantoms are visualized in Figure 2 (right). After digitization, 108 124 373 coincidences were recorded.

### 2.6 Real Scanner 1—semi‐monolithic crystal slabs

**FIGURE 3:**
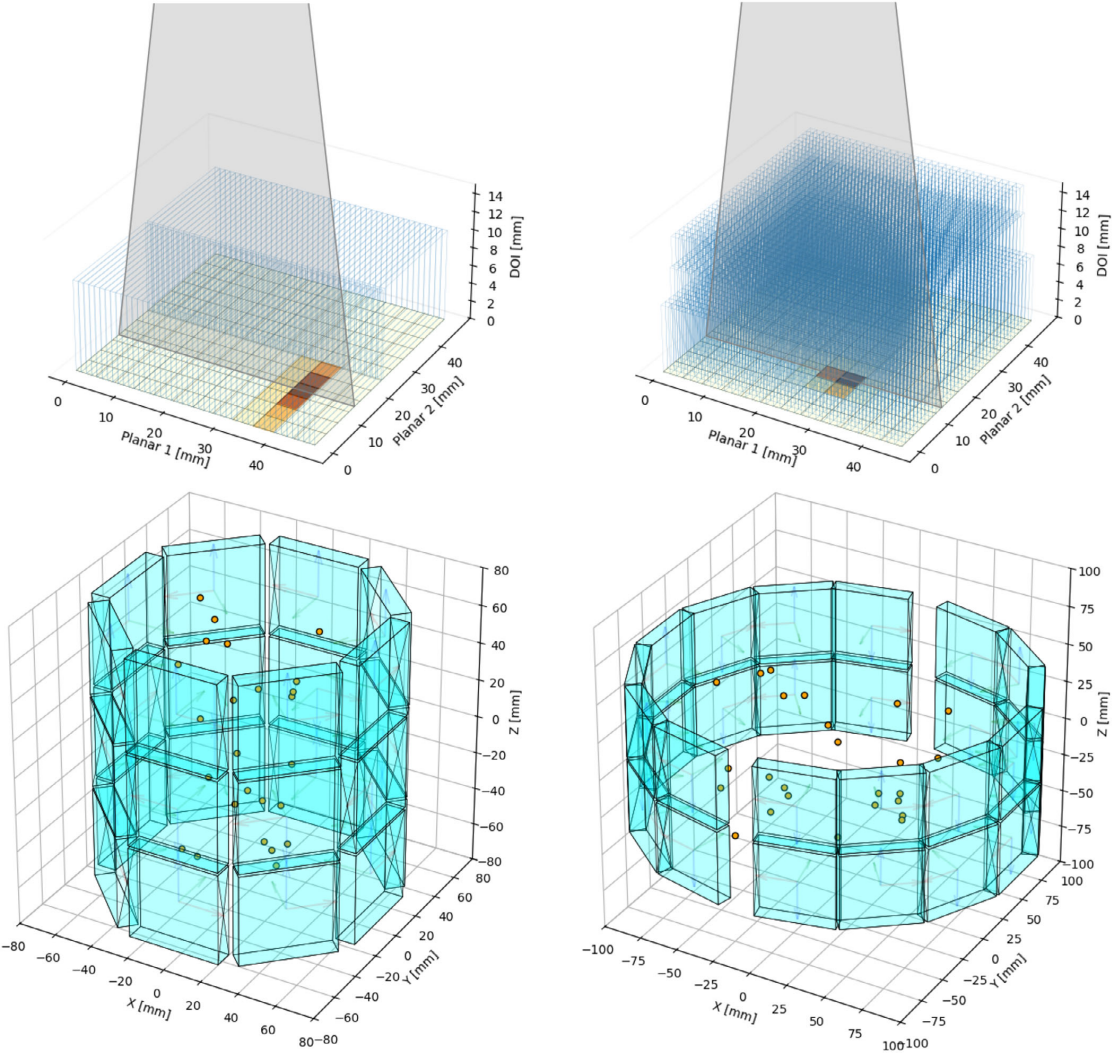
Detector geometries (top) with fan‐beam irradiation setup (gray), crystal outlines (blue), lightspreads (orange), and corresponding scanner blueprints (bottom) for both real systems used in this work. Scanner No. 1 (left) uses two semi‐monolithic slab arrays, while scanner no. 2 (right) relies on a multi‐layer segmented crystal setup. Both blueprints include 25 randomly selected point sources (orange) used for alignment calibration.

We also applied our method to a real scanner assembled by our group. This scanner likewise features 24 detector blocks arranged in 3 rings. We employ a finely segmented semi‐monolithic detector design,^20^ comprising two slab arrays, each consisting of 44 LYSO slabs with dimensions of $1\times 24\times 10\;mm^{3}\;$, surrounded on their side surfaces by a 0.1‐mm ${\mathrm{BaSO}}_{4}$ layer, cf. Figure 3 (left). Readout is performed using a 12×12 array of SiPM channels, realized through a 6×6 grid of Philips DPC sensors, each providing 2×2 digital channels.^21^ These sensors are mounted on the Hyperion DPC‐Tile circuit board, developed by Hyperion Hybrid Imaging Systems.^22^

In a benchtop calibration setup, we collected a dataset of fan‐beam irradiations in all three spatial dimensions for each detector block. We used this dataset to train gradient boosted tree models (GTB),^23^, ^24^ with which we achieve a mean absolute error in gamma‐interaction‐positioning of ∼1mm in all three spatial dimensions. We used a time‐walk calibration to achieve a CTR of approximately 500ps. Crystal efficiencies were computed from a cylindrical shell measurement of 464 167 434 coincidences over 14 426s, using the fan‐sum method where each detector is in coincidence with three opposing detectors, as well as the axially shifted counterparts above and below, resulting in a total of nine coincident detectors, cf. Figure 1 (right). For a detailed description of the detectors and the applied position, time, and energy calibration setup, refer to the works by Kuhl et al.^12^, ^20^ and Schug et al.^25^

The mechanical alignment of the detectors within the support and cooling structures was ensured by kinematic mounting. The detector modules were assembled using precision‐located recesses, while custom‐aligned bonding of the crystals and sensor interfaces minimized structural tolerances. All mechanical components were referenced to a common, precisely machined gantry. These efforts resulted in a scanner with exceptionally well‐aligned detector positions. As thus only small deviations of the blueprint from the ground‐truth alignment are expected, we introduce artificial misalignment by perturbing the blueprint configuration with Gaussian noise ($\sigma_{T}=2\;mm,\sigma_{R}=2^{\circ }$). This randomized blueprint serves as the initialization for our optimization. The original blueprint and the randomized alignment, are shown in Figure 5 (top). We utilized a 3D linear motor stage to acquire a dataset using a 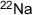 point source, systematically moved within the scanner along a fine grid of positions.^20^ The measurement time per position was 3.1s. To evaluate our optimization approach, we randomly sampled 25 positions within the scanner, cf. Figure 3 (left). This selection resulted in the acquisition of 5 957 886 coincidences.

**FIGURE 4:**
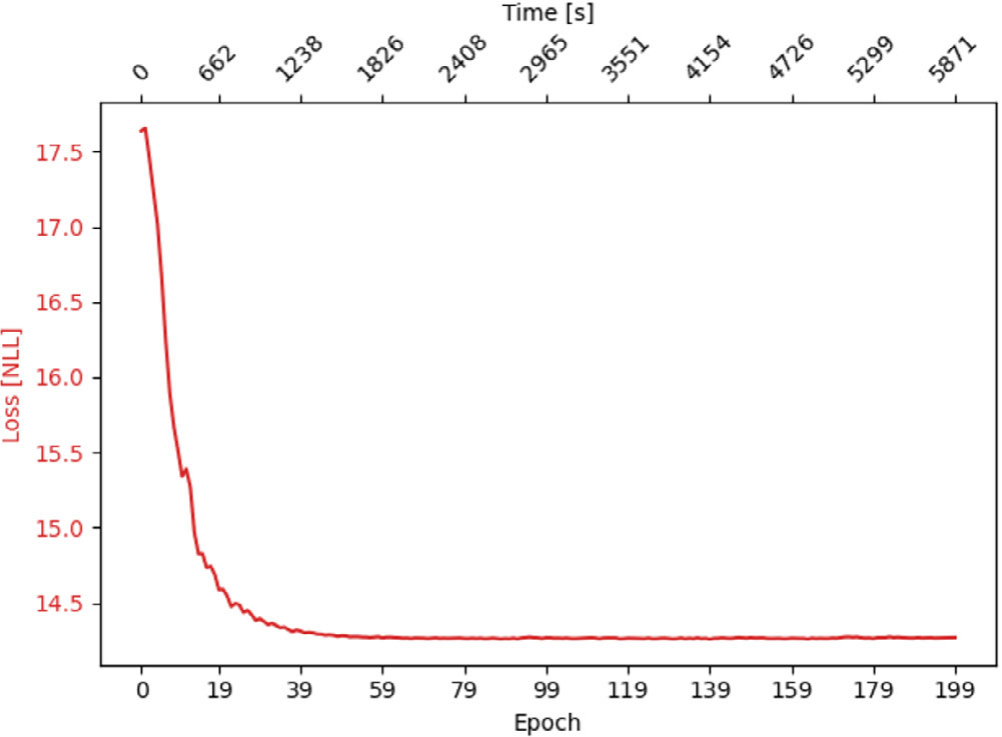
Evolution of the negative log‐likelihood (NLL) loss over optimization epochs for simulated point‐source data, with the optimization performed using TOF information.

**FIGURE 5:**
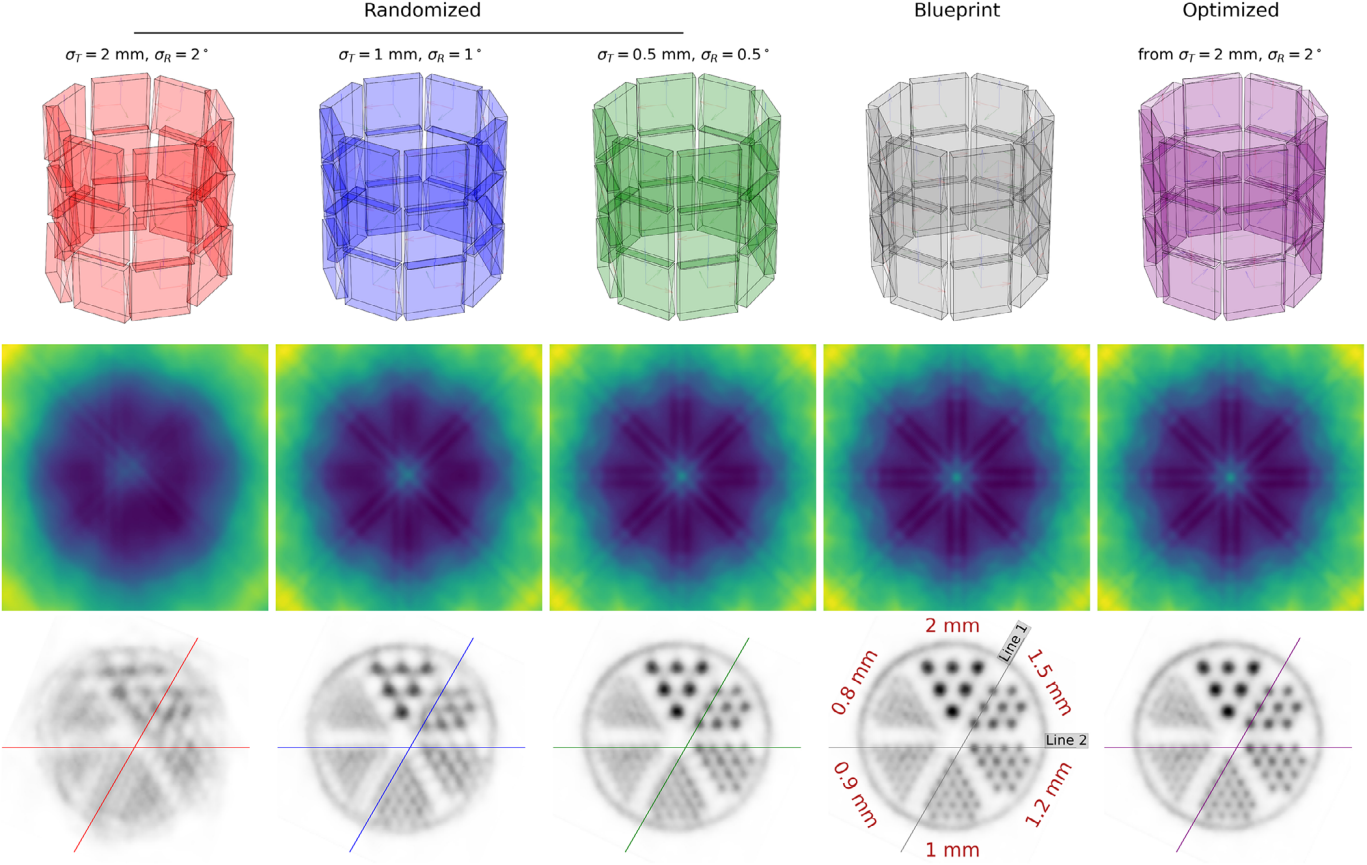
Scanner no. 1 configurations (top), sensitivity maps (middle), and image reconstructions with $0.2\times 0.2\times 0.2\;mm^{3}$ voxel size (bottom) for the misaligned scanners, the blueprint, and the optimized scanner. Reconstructions were performed using 500 million coincidences within a 450keV–650keV energy window. The initial activity of the phantom scan was ∼5MBq.

Imaging performance was evaluated using a HotRod image quality phantom filled with 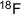
‐FDG, featuring rods with diameters of 2, 1.5, 1.2, 1.0, 0.9, and 0.8mm. The phantom had a starting activity of 5.5MBq, and 500 million coincidences were collected after 4598s. These coincidences were used for the reconstruction, split into 10 subsets using an equal transaxial angular distribution per subset. 10 LORs per coincidence event were backprojected by sampling inside the crystal voxels.

### 2.7 Real scanner 2—multi‐layer segmented crystals

Besides the semi‐monolithic setup, we also assessed our method using a different scanner prototype developed by our group, featuring a multi‐layer segmented crystal design. This scanner comprises 28 detector blocks arranged in 2 rings, where each ring consists of two offset half‐rings, cf. Figure 3 (right). The detectors use the same 12×12 SiPM readout architecture based on the Philips DPC sensors on Hyperion DPC‐Tile boards. Each detector comprises three arrays of needle‐shaped LYSO crystals arranged in a staggered structure for depth‐of‐interaction (DOI) encoding. The segments were separated by ${\mathrm{BaSO}}_{4}$ with a thickness of 0.085,mm. The arrays share identical transverse dimensions of 1.3mm × 1.3mm, while their heights progressively decrease from bottom to top: 7.4mm (bottom array, near SiPMs), 4.4mm (middle array), and 3.2mm (top array). Each array is laterally offset by half a crystal pitch relative to the array below.

For calibration, a fan‐beam irradiation dataset was again acquired; this time, however, it was limited to a single detector block for which fan‐beam irradiations were performed for all three spatial dimensions. Gamma interaction positions were calibrated using GTB models, independently trained for each spatial dimension. We used a time‐walk calibration^25^ to achieve a CTR of approximately 500ps. Crystal efficiencies were again determined via the fan‐sum method, utilizing a measurement of 30 968 765 coincidences of a point source moved along the axial center of the scanner for 7938s, accepting coincidences of each detector with both its opposing half‐rings.

As before, we evaluated our alignment optimization by perturbing the blueprint geometry with Gaussian noise ($\sigma_{T}=2\;mm$, $\sigma_{R}=2^{\circ }$). Perturbations were applied both individually for each detector and collectively for the combined sets of the left two half‐rings and the right two half‐rings. We moved a 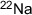 point source across a large 3D grid within the scanner's FOV. The measurement time per position was 8s. From this dataset, 25 source positions were randomly selected for evaluation, resulting in the digitization of 4 413 210 coincidences used for the optimization, cf. Figure 3 (right).

To evaluate imaging performance, we created a HotRod‐like phantom by scanning a 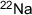 point source attached to a motorized stage. Rod diameters range from 2mm down to 0.8mm, analogous to the 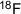
‐FDG phantom used for scanner no. 1. A total of 75 million coincidences were recorded after 10561s, split into 20 subsets using an equal transaxial angular distribution per subset. 100 LORs per coincidence event were backprojected by sampling inside the crystal voxels.

All collected data, for simulation as well as real scanners, was filtered to the energy range of 450keV–650keV.

### 2.8 Evaluation metrics

For the simulations, we performed optimization using a cylindrical configuration as initial starting point. For the real scanners, we used the noised configurations ($\sigma_{T}=2\;mm,\sigma_{R}=2^{\circ }$) as initial starting point. As the motor stage/ phantom might in practice be misplaced inside the scanner, we performed a Procrustes analysis to first map the found configuration back onto the ground‐truth by means of a global rotation and translation. This provided a means to assess how well the detector blocks are aligned relative to one another. We then evaluated the error of found rotations $\Delta R_{i}$ and translations $\Delta T_{i}$ across all detectors for each dimension i. These were computed as follows, where $A_{d,i}$ represents an axis vector of detector d for dimension i:

(6) $$\begin{array}{cc} \Delta T_{i} & =\frac{1}{N_{\text{det}}}\sum_{d=1}^{N_{\text{det}}}\left|P_{d,i}^{*}-{\hat{P}}_{d,i}\right| \end{array}$$

(7) $$\begin{array}{cc} \Delta R_{i} & =\frac{1}{N_{\text{det}}}\sum_{d=1}^{N_{\text{det}}}\arccos\left(\frac{\left\langle A_{d,i}^{*},{\hat{A}}_{d,i}\right\rangle }{∥A_{d,i}^{*}∥∥{\hat{A}}_{d,i}∥}\right). \end{array}$$

For the real scanners, we additionally performed 10 iterations of a List‐Mode Ordered Subsets Expectation Maximization (LM‐OSEM) image reconstruction.^14^ We used a voxel size of $0.2\times 0.2\times 0.2\;mm^{3}$ for the reconstruction. For the different alignment configurations, we computed sensitivity maps using the attenuation map A and detection efficiencies $E_{d,cr}$, recomputed after alignment optimization and including geometric factors. The attenuation maps additionally included the LYSO crystals to directly account for block‐geometrical effects. During both, normalization and reconstruction, we sampled random positions inside both crystal voxels, using 10 samples per LOR to mitigate discretization artifacts. We utilized a TOF full‐width‐half‐maximum (FWHM) of 75mm, corresponding to the CTR of 500ps. For scanner no. 1 we performed reconstructions with various amounts of noise, that is, ($\sigma_{T}=2\;mm,\sigma_{R}=2^{\circ }$), ($\sigma_{T}=1\;mm,\sigma_{R}=1^{\circ }$), and ($\sigma_{T}=0.5\;mm,\sigma_{R}=0.5^{\circ }$), as well as for the blueprint and the optimized configuration. For scanner no. 2, we performed reconstructions for noise applied to individual detectors ($\sigma_{T}=2\;mm,\sigma_{R}=2^{\circ }$), noise applied to the groups of the half rings with ($\sigma_{T}=2\;mm,\sigma_{R}=2^{\circ }$), as well as for the blueprint and the optimized detector individual and half‐ring configurations. Each reconstructed image was aligned to the ground‐truth using separately fitted affine transformations before evaluation. We evaluated reconstructed images using the peak‐signal‐to‐noise ratio (PSNR) and structural similarity index (SSIM), given by:

(8) $$\begin{array}{cc} \text{PSNR} & =10\cdot \log_{10}\left(\frac{\text{MAX}{\left(I\right)}^{2}}{\text{MSE}(I,\hat{I})}\right) \end{array}$$

(9) $$\begin{array}{cc} \text{SSIM} & =\frac{\left(2\mu_{I}\mu_{\hat{I}}+C_{1}\right)\left(2\sigma_{I\hat{I}}+C_{2}\right)}{\left(\mu_{I}^{2}+\mu_{\hat{I}}^{2}+C_{1}\right)\left(\sigma_{I}^{2}+\sigma_{\hat{I}}^{2}+C_{2}\right)} \end{array}$$

where I and $\hat{I}$ denote the reference and reconstructed images, respectively. $\mu_{I}$ and $\mu_{\hat{I}}$ are the mean intensities, $\sigma_{I}^{2}$ and $\sigma_{\hat{I}}^{2}$ the variances, and $\sigma_{I\hat{I}}$ the covariance. $C_{1}$ and $C_{2}$ are small stabilization constants. For noise uniformity, we additionally report the coefficient of variation (COV) inside a uniformly filled phantom region. A binary mask Ω is derived from the ground‐truth phantom to select the uniform area, and COV is computed as

(10) $$\text{COV}=100\times \frac{\sigma_{\Omega }\left(\hat{I}\right)}{\mu_{\Omega }\left(\hat{I}\right)},$$

where $\mu_{\Omega }\left(\hat{I}\right)$ and $\sigma_{\Omega }\left(\hat{I}\right)$ denote the mean and standard deviation of the reconstructed activity within Ω, respectively (reported in %). Additionally, we inspected line profiles and assessed the peak‐to‐valley ratio (PVR) by averaging the values across all line profiles corresponding to each rod diameter. For scanner no. 2, we omitted quantitative image analysis due to the nature of the underlying phantom, which was generated using a 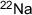 point source. Owing to the spherical geometry of the source, activity distribution toward the edges of the rods is not sharply defined but gradually increases, leading to non‐uniform rod filling. We therefore restricted our evaluation to a qualitative visual assessment.

## 3 RESULTS

### 3.1 Simulated point sources

Alignment results for the simulated point sources are given in Table 1 with and without the usage of TOF. We can observe that deviations from the ground truth up to 1.95mm and 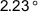 are present in the randomized initial configuration. Optimization using TOF information improves results for most crystal binning configurations. Worst results are achieved for the coarser crystal binnings without DOI information, with dimension‐wise mispositionings up to ∼ 215μm and misorientations of 1.09
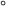
–2.24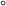. For the *y*‐orientation, performance is worse compared to the initialization. Usage of higher crystal binning resolutions, especially in DOI direction, greatly improves results. For the $2\times 2\times 2\;mm^{3}$ and $1\times 1\times 1\;mm^{3}$ case results are very similar and in the range of mispositionings of 40μmto50μm and misorientations of 0.11
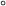
–0.23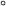, indicating an excellent agreement with the ground‐truth configuration. The convergence behavior of the optimization is shown in Figure 4. The negative log‐likelihood (NLL) loss exhibits a rapid decrease within the first ∼40 epochs, after which convergence is achieved with only minor fluctuations. The complete optimization required approximately 1h and 38min on a single GPU.

**TABLE 1:** Alignment parameter deviations from GT for all simulation setups.

				Optimized (various crystal binnings / $\;mm^{3}$)			
			Initialization cylindrical	3×3×10	2×2×10	2×2×2	1×1×1
Point Sources	(no TOF)	$\Delta T_{x}$ (mm)	1.9519	0.3280	0.2398	0.0802	**0.0560**
		$\Delta T_{y}$ (mm)	1.3649	0.2025	0.1474	0.0639	**0.0524**
		$\Delta T_{z}$ (mm)	1.4681	0.1542	0.0973	0.0599	**0.0586**
		$\Delta R_{x}$ (∘)	1.73	1.35	0.98	0.19	**0.11**
		$\Delta R_{y}$ (∘)	1.16	2.10	1.65	0.23	**0.14**
		$\Delta R_{z}$ (∘)	2.23	1.63	1.32	0.16	**0.11**
Point Sources		$\Delta T_{x}$ (mm)	1.9519	0.1866	0.1428	0.0465	**0.0379**
		$\Delta T_{y}$ (mm)	1.3649	0.2141	0.1725	0.0481	**0.0400**
		$\Delta T_{z}$ (mm)	1.4681	0.2076	0.1046	0.0456	**0.0445**
		$\Delta R_{x}$ (∘)	1.73	1.09	0.67	0.18	**0.13**
		$\Delta R_{y}$ (∘)	1.16	2.24	2.00	0.23	**0.14**
		$\Delta R_{z}$ (∘)	2.23	2.02	1.84	0.16	**0.11**
Tube Phantom		$\Delta T_{x}$ (mm)	1.9519	0.6127	0.4965	0.1931	**0.1193**
		$\Delta T_{y}$ (mm)	1.3649	0.3175	0.2510	0.0871	**0.0521**
		$\Delta T_{z}$ (mm)	1.4681	0.3371	0.2765	0.1314	**0.0796**
		$\Delta R_{x}$ (∘)	1.73	1.04	0.88	0.22	**0.14**
		$\Delta R_{y}$ (∘)	1.16	1.48	1.33	0.33	**0.20**
		$\Delta R_{z}$ (∘)	2.23	0.95	0.91	0.25	**0.15**

*Note*: Bold numbers indicate the best result per row.

### 3.2 Simulated tube phantom

For the simulated tube phantom, we observe a similar trend: a higher gamma‐positioning resolution leads to improved alignment performance, as shown in Table 1 (bottom). The worst positioning errors now range from approximately 318–613μm, with misorientations between 0.95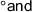 1.48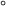. In contrast, the finest crystal binning resolutions achieve positioning errors of around 52μmto193μm and misorientations between 0.14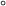 and 0.33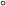. Overall, performance is reduced compared to the point source simulation, likely due to the increased complexity of the dense distribution matching task, which may require more data to effectively resolve spatial ambiguities.

### 3.3 Real point sources—scanner no. 1

**FIGURE 6:**
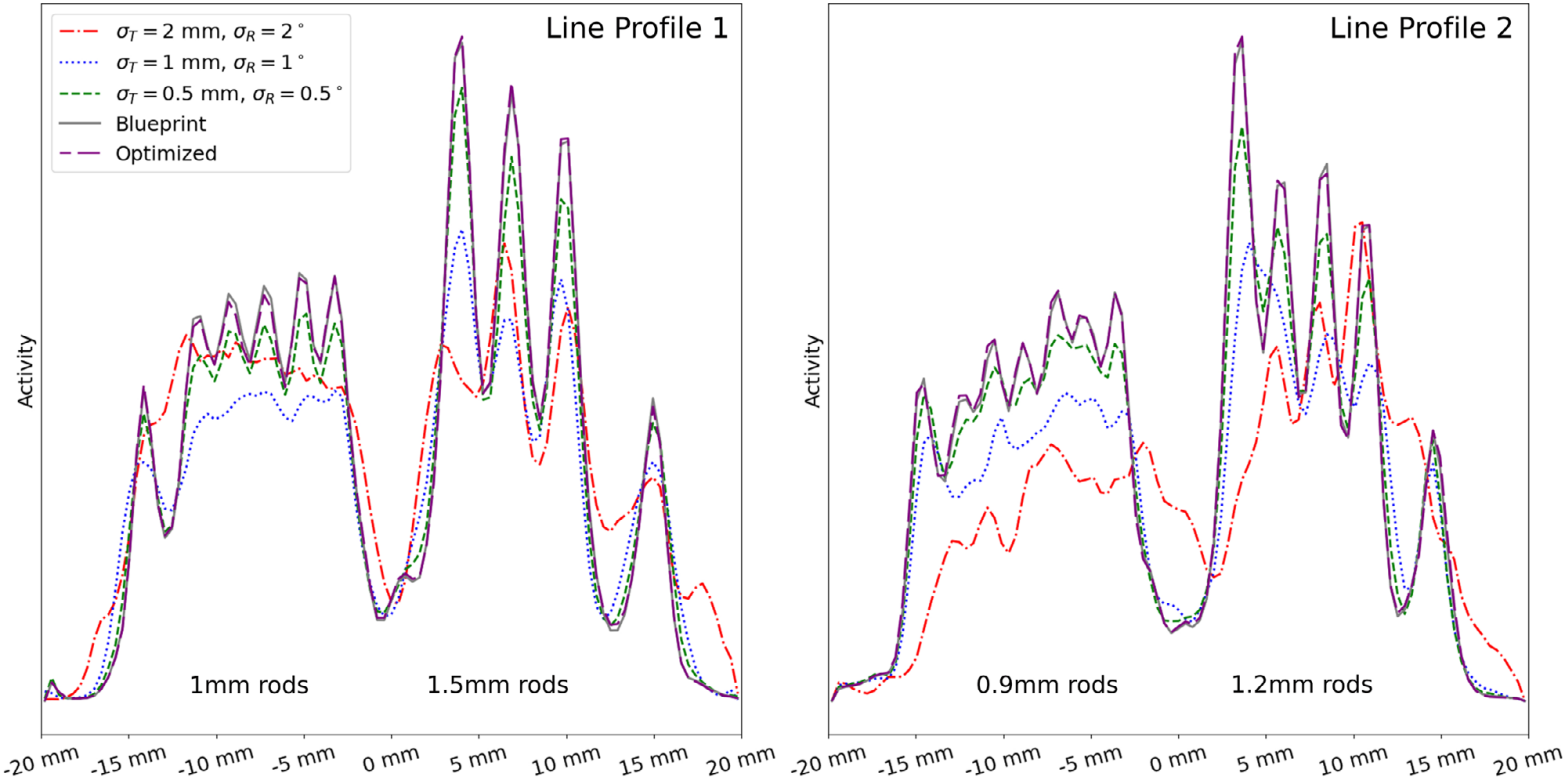
Line profiles for scanner no. 1. The first and last peaks correspond to the filled shell surrounding the rod chambers. Reconstructions are sum‐normalized; the *y*‐axis represents normalized counts/activity.

The randomized alignment configurations, the original blueprint, as well as the optimization result can be inspected in Figure 5 (top). Alignment results are given in Table 2. The optimized configurations closely follows the predefined blueprint. For coarse crystal binning, alignment errors are approximately 400μm and 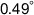
–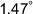, while the finest binning achieves maximal errors of 145μm and 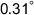. The slightly better performance observed for the $2\times 2\times 2\;mm^{3}$ configuration compared to $1\times 1\times 1\;mm^{3}$ may be attributed to bias effects introduced by the positioning models. Reconstructed images (cf. Figures 5 and 6), using the $1\times 1\times 1\;mm^{3}$ binning optimization results, show that both the blueprint and the optimized scanner achieve separability of the 1mm rods, supported by PVRs above 1.3, given in Table 3. In contrast, the heavily randomized initial alignment results in severely blurred reconstructions, where the 1.2mm rods are no longer distinguishable. However, all randomizations visually reduce image quality compared to the blueprint and optimized configurations. The line profile plot in Figure 6 supports this observation. PSNR, SSIM, and COV values are given in Table 4. Blueprint and optimized configurations result in near‐identical results across all metrics, with the optimized configuration showing a slight performance edge. In contrast, increasing misalignment results in a marked degradation for all cases. The aforementioned gamma positioning bias effects manifest in the sensitivity maps as stripe‐like patterns extending from the center toward the central interface of the slab arrays within each detector.

**TABLE 2:** Alignment parameter deviations from blueprint for real scanner no. 1.

	Initialization	Optimized (various crystal binnings /$\;mm^{3}$)			
	randomized	3×3×10	2×2×10	2×2×2	1×1×1
$\Delta T_{x}$ (mm)	1.7619	0.4474	0.4049	**0.1418**	0.1451
$\Delta T_{y}$ (mm)	1.5156	0.3747	0.0954	0.0967	**0.0935**
$\Delta T_{z}$ (mm)	1.4881	0.3688	0.3512	**0.1323**	0.1366
$\Delta R_{x}$ (∘)	2.38	0.49	0.31	**0.21**	0.23
$\Delta R_{y}$ (∘)	2.64	1.55	0.74	0.32	**0.31**
$\Delta R_{z}$ (∘)	2.66	1.47	0.72	0.31	**0.28**

*Note*: Bold numbers indicate the best result per row.

**TABLE 3:** Peak‐to‐Valley ratios for scanner no. 1 for all evaluated configurations across rod diameters.

Configuration	2.0 mm	1.5 mm	1.2 mm	1.0 mm	0.9 mm	0.8 mm
$\sigma_{T}=2$ mm, $\sigma_{R}=2^{\circ }$	1.3971	1.3232	1.2335	1.0885	**1.1635**	1.0904
$\sigma_{T}=1$ mm, $\sigma_{R}=1^{\circ }$	2.6864	1.4497	1.1211	1.1131	1.0892	1.0591
$\sigma_{T}=0.5$ mm, $\sigma_{R}=0.5^{\circ }$	3.4523	1.8700	1.3800	1.1758	1.0866	1.0704
Blueprint	3.9527	2.1473	1.5831	1.3097	1.1442	**1.1071**
Optimized	**4.0064**	**2.1673**	**1.5961**	**1.3128**	1.1388	1.1069

*Note*: Bold numbers indicate the best result per column.

**TABLE 4:** PSNR, SSIM, and COV values of reconstructed images of scanner no. 1 for the different alignment configurations.

Configuration	PSNR (dB)	SSIM	COV (%)
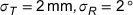	14.0331	0.3407	11.11
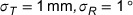	14.3238	0.3918	5.79
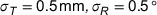	14.5012	0.4055	5.33
Blueprint	14.5963	0.4125	5.29
Optimized	**14.6016**	**0.4125**	**5.24**

*Note*: Bold numbers indicate the best result per column.

Abbreviation: COV, coefficient of variation; PSNR, peak signal‐to‐noise ratio; SSIM, structural similarity index.

### 3.4 Real point sources—scanner no. 2

**FIGURE 7:**
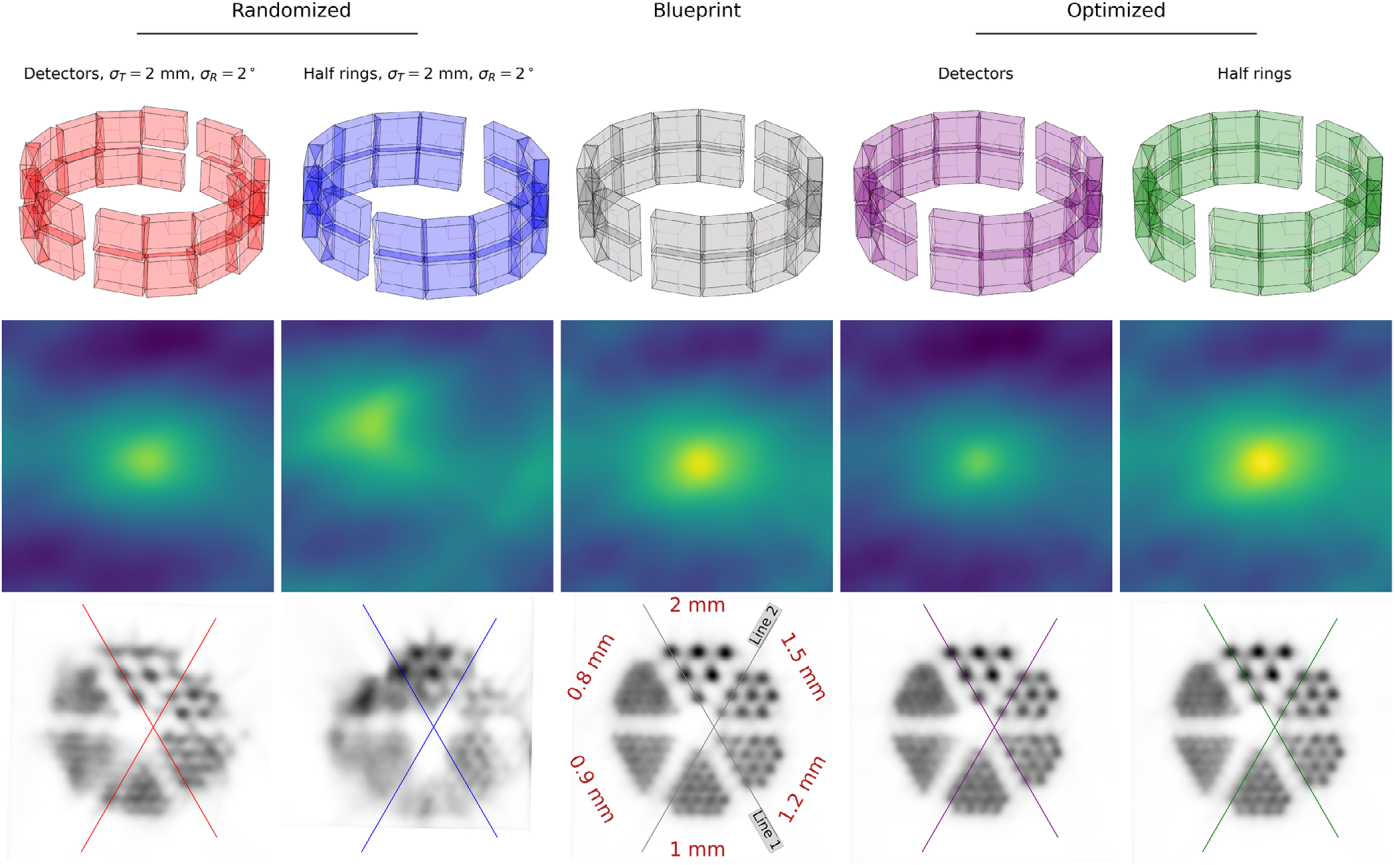
Scanner no. 2 configurations (top), sensitivity maps (middle), and image reconstructions with $0.2\times 0.2\times 0.2\;mm^{3}$ voxel size (bottom) for the misaligned scanners, the blueprint, and the optimized scanners. Reconstructions were performed for a hotRod dose‐painting using a 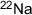 point source mounted on a linear motor stage. A total of 500 million coincidences within an energy window of 450keV were used.

**FIGURE 8:**
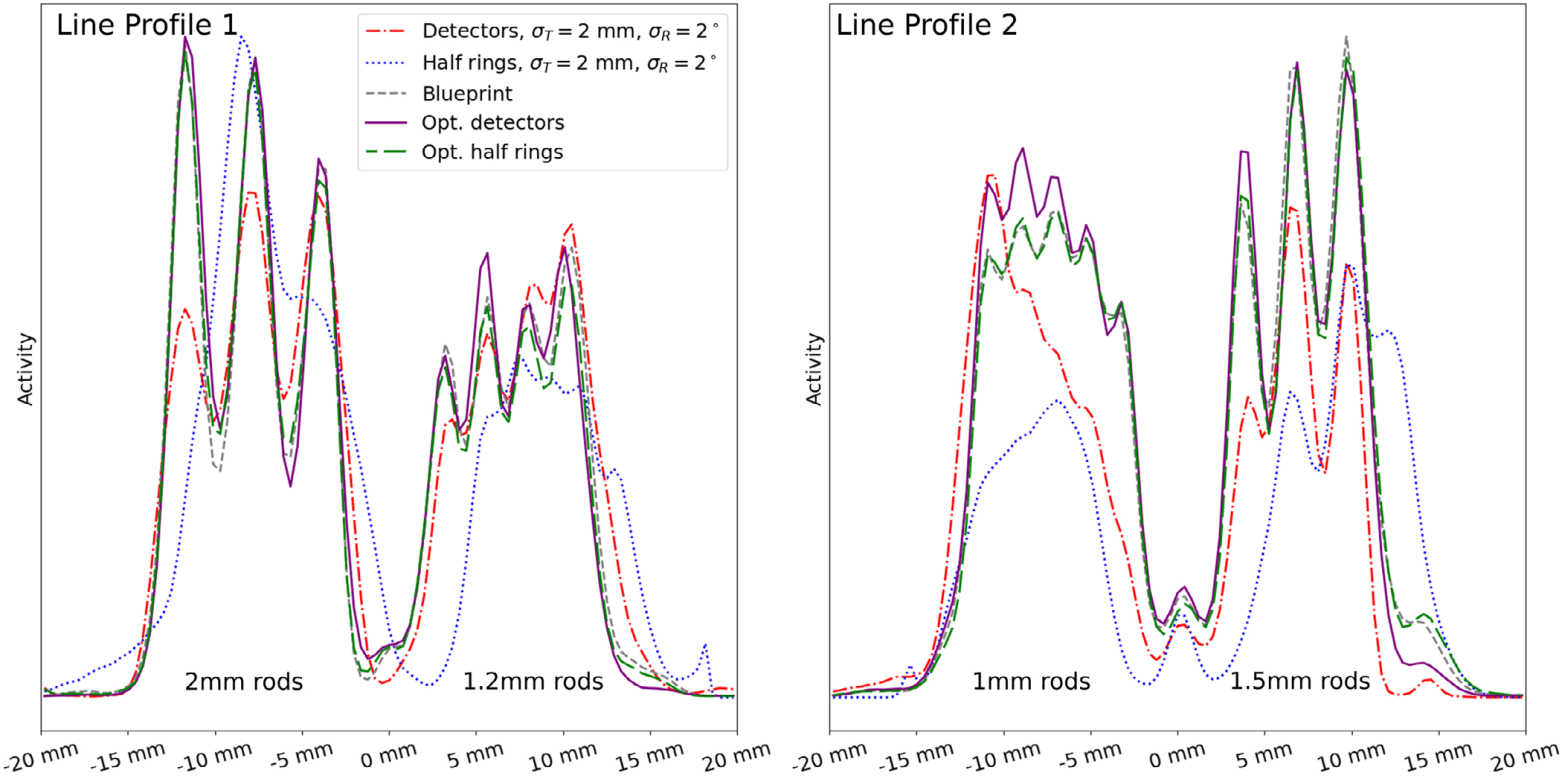
Line profiles for scanner no. 2. Reconstructions are sum‐normalized; the y‐axis represents normalized counts/activity.

Figure 7 shows scanner configurations and reconstructed images for both randomized, the blueprint, and the optimized alignment configurations of the second scanner. Alignment results compared to the blueprint are given in Table 5. For the detector individual optimization, alignment parameters approach the blueprint primarily in the *y*‐translation and *z*‐rotation, indicating close agreement in the transaxial plane. In contrast, axial parameters show a notable deviation. The reconstructed images of the optimized configurations exhibit almost indistinguishable quality. For both randomizations, the image quality is severely degraded. After optimization, both the half‐ring and detector‐individual configurations recover visual rod separability down to 1.2mm, comparable to the blueprint. The line profiles shown in Figure 8 support this observation. Among the two, the detector‐individual optimization yields visibly slightly cleaner reconstructions, with less spurious activity between the triangular rod patterns. The sensitivity maps reveal pronounced differences between configurations; more symmetrical setups—such as the blueprint and optimized half rings—exhibit the highest sensitivity maxima.

**TABLE 5:** Alignment parameter deviations from blueprint for real scanner no. 2.

	rand. (detectors)	opt. (detectors)	rand. (half‐rings)	opt. (half‐rings)
$\Delta T_{x}$ (mm)	1.5521	2.2616	5.6767	1.3573
$\Delta T_{y}$ (mm)	1.4906	0.4679	3.2760	0.1532
$\Delta T_{z}$ (mm)	1.5504	2.0139	4.1664	1.6285
$\Delta R_{x}$ (∘)	2.19	2.25	3.05	1.17
$\Delta R_{y}$ (∘)	1.92	2.37	3.05	1.17
$\Delta R_{z}$ (∘)	2.22	1.41	2.40	0.30

*Note*: Optimization is performed for individual detectors as well as half‐rings.

## 4 DISCUSSION

Our findings highlight the critical importance of precise scanner alignment in high‐resolution PET imaging. Even minor deviations, such as $\sigma_{T}=0.5\;mm$ in translation and 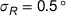 in rotation, lead to observable degradation in image quality, as also reflected by a PSNR decrease of approximately 0.99% and a COV decrease of 0.98%, potentially compromising diagnostic accuracy and negatively impacting patient outcomes.

Our results demonstrate that maximum likelihood alignment estimation is effective for both point sources and phantoms with more complex tracer distributions. For point sources, we observe robustness even with limited data availability, as evidenced by the successful optimization of the real PET scanners using only ∼6 million and ∼4.4 million coincidences. The successful alignment using the tube phantom further confirms that the procedure can be performed using dedicated calibration phantoms, eliminating the need for a motorized stage setup. Optimization with real data demonstrates that 1mm image quality is achievable even without a precise blueprint. Phantom misalignment relative to the global scanner coordinate system does not affect performance, as the correct relative alignment is still recovered, verified by performing a Procrustes analysis before evaluation. The optimization for scanner no. 2 additionally demonstrates, that alignment optimization can be successfully performed for more complex crystal topologies and even under model‐sharing conditions, where only one detector block is fan‐beam calibrated. Also, the results show that the method can be applied to entire sets of detectors with shared learnable parameters. While we used the Adam optimizer for all experiments, the method is compatible with any differentiable optimizer and can be adapted to suit the specific characteristics of the calibration task at hand.

Higher crystal binning resolutions tend to reduce alignment errors, especially when DOI information is utilized. Several other factors influence alignment performance, including voxel size and energy filtering. Preliminary tests, not included in this manuscript, indicate that larger voxel sizes tend to degrade performance; however, a systematic ablation study is needed to precisely quantify individual contributions of these variables. The design of the tracer distribution used for the calibration measurement also plays a critical role for successful alignment optimization. Distributions that are too simple may lack the spatial coverage or variability needed to constrain all detector parameters, while overly complex phantoms may demand prohibitively large datasets to effectively handle the resulting dense distribution matching task. Tracer distributions near the center of the FOV provide low geometric leverage: they constrain many detectors, but the resulting constraints are weak and ambiguous due to symmetry. In contrast, activity near the periphery offers high geometric leverage, sharply constraining a smaller subset of detectors and enabling precise local refinement. Broad distributions spanning the entire FOV balance both effects, supporting global alignment consistency. Furthermore, non‐uniformity of the phantom filling, for example, due to air bubbles, might play an important role that needs to be considered for phantom design.

Regarding scalability to larger scanners, we point out that an increased FOV necessitates larger voxel grids, leading to substantial GPU memory demands with small voxel sizes due to cubic scaling. Ideas for efficient memory handling, possibly involving adaptive voxel grids only selectively loaded into memory, should be addressed in future research.

Future work will focus on extending the proposed framework to estimate intra‐block misalignments at the level of individual crystals. This will allow us to determine whether residual errors persist within detector blocks or are already fully compensated for by the gamma positioning calibration. Furthermore, the robustness of the method against severe geometric perturbations and potential detector mislabelings will be systematically investigated. Additional experiments are planned to explore adaptive hierarchical calibration schemes, where coarse block alignment is followed by fine‐grained crystal‐level optimization. Finally, we aim to validate the approach on clinical systems, further assessing both stability and convergence behavior across a broader range of scanner geometries and calibration phantoms.

The proposed optimization framework relaxes the mechanical precision requirements for PET scanner assembly, offering the potential to reduce manufacturing complexity and cost. By enabling in‐system calibration of detector alignment parameters, it compensates for assembly tolerances and ensures accurate LOR positioning. The method also supports routine recalibration without external equipment, making it particularly valuable for mobile PET systems and environments subject to mechanical stress or frequent configuration changes. In addition, it simplifies module replacement and supports seamless integration of future hardware upgrades.

## 5 CONCLUSIONS

Our study demonstrates that robust detector alignment calibration can be performed directly from measurement data using a maximum likelihood‐based optimization. Calibration with both point sources and a tube phantom, as well as different crystal topologies, validates the method's capacity to handle complex phantom definitions and various scanner setups. For the real scanners, despite significant initial misalignment and the absence of precise phantom–scanner alignment, the optimization achieves image quality on par with a precise blueprint, enabling high‐quality image reconstruction while allowing for more relaxed manufacturing tolerances through efficient in‐system recalibration. Altogether, the approach offers a practical and scalable path to precise PET detector alignment, driven not by mechanical perfection but by the statistical structure of the measured data.

## CONFLICT OF INTEREST STATEMENT

The authors declare no conflicts of interest.
